# Urine proteomics for profiling of mouse toxoplasmosis using liquid chromatography tandem mass spectrometry analysis

**DOI:** 10.1186/s13071-021-04713-6

**Published:** 2021-04-20

**Authors:** Lin-Lin Cui, Chun-Xue Zhou, Bing Han, Sha-Sha Wang, Si-Ying Li, Shi-Chen Xie, Dong-Hui Zhou

**Affiliations:** 1 Key Laboratory of Fujian-Taiwan Animal Pathogen Biology, College of Animal Sciences (College of Bee Science), Fujian Agriculture and Forestry University, Fuzhou, 350002 Fujian China; 2 Department of Pathogen Biology, School of Basic Medical Sciences, Cheeloo College of Medicine, Shandong University, Jinan, Shandong Province 250012 People’s Republic of China; 3 College of Veterinary Medicine, Northwest A&F University, Yangling, Shaanxi 712100 People’s Republic of China; 4 State Key Laboratory of Veterinary Etiological Biology, Key Laboratory of Veterinary Parasitology of Gansu Province, Lanzhou Veterinary Research Institute, Chinese Academy of Agricultural Sciences, Lanzhou, Gansu Province 730046 People’s Republic of China

**Keywords:** *Toxoplasma gondii*, Urine, Proteomics, Biomarkers

## Abstract

**Background:**

*Toxoplasma gondii* is an obligate intracellular parasite that causes toxoplasmosis. Urine is an easily obtained clinical sample that has been widely applied for diagnostic purposes. However, changes in the urinary proteome during *T. gondii* infection have never been investigated.

**Methods:**

Twenty four-hour urine samples were obtained from BALB/c mice with acute infection [11 days post infection (DPI)], mice with chronic infection (35 DPI) and healthy controls, and were analyzed using a label-free liquid chromatography tandem mass spectrometry analysis.

**Results:**

We identified a total of 13,414 peptides on 1802 proteins, of which 169 and 47 proteins were significantly differentially expressed at acute and chronic infection phases, respectively. Clustering analysis revealed obvious differences in proteome profiles among all groups. Gene ontology analysis showed that a large number of differentially expressed proteins (DEPs) detected in acute infection were associated with biological binding activity and single-organism processes. KEGG pathway enrichment analysis showed that the majority of these DEPs were involved in disease-related and metabolic pathways.

**Conclusions:**

Our findings revealed global reprogramming of the urine proteome following *T.* *gondii* infection, and data obtained in this study will enhance our understanding of the host responses to *T. gondii* infection and lead to the identification of new diagnostic biomarkers.

**Graphic Abstract:**

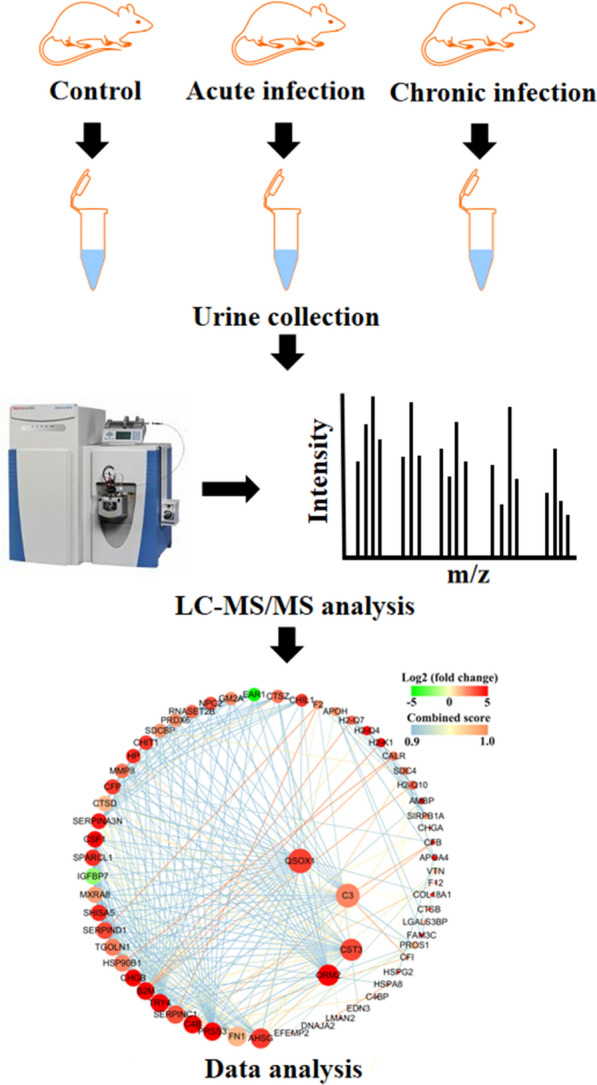

**Supplementary Information:**

The online version contains supplementary material available at 10.1186/s13071-021-04713-6.

## Background

*Toxoplasma gondii* was first found in hamster-like rodents *Ctenodactylus gundi* in Tunisia in the early twentieth century. It is now a globally distributed apicomplexan protozoan that can infect almost all warm-blooded animals and humans and cause zoonotic toxoplasmosis [1]. About one third of the world's human population carries this parasite, but very few have symptoms. However, unborn children may have diseases of the nervous system and eyes if their mothers are infected with *Toxoplasma*. Immunocompromised individuals, such as HIV carriers and organ transplant patients, may experience severe symptoms [2]. Up to now, no commercial vaccines and drugs are available which can completely eliminate this parasite from the infected human body.

Infection occurs when warm-blooded animals eat food or water contaminated with *Toxoplasma* oocysts or cysts [3]. Parasite will invade and rapidly proliferate in the intestinal epithelial cells and then disseminate to virtually all parts of the human body. Once parasites are controlled by a potent host immune response, they will differentiate into the bradyzoite encysted in tissue cyst in muscles and brain. A series of pathological changes in the internal organs in animal models and humans have been largely reported, such as encephalitis, splenomegaly, myocarditis and hydrocephalus [4]. Pathological changes result from tissue destruction which induce substantial changes in protein metabolism and will alter the amount of peptides in the bloodstream. These peptides will be further processed by the kidneys and possibly present in the urine. In addition, the kidney is found severely affected during acute toxoplasmosis [5].

Compared with other body fluid, urine is stable and easily obtained non-invasively in large quantities. Proteomics is an important post-genomic approach that is used to determine the entire set of proteins of an organism or biological system. Characterizing a proteome can help to understand the pathological and physiological changes in a biological system and offer valuable insights on the development and progression of disease [6]. Urine has become one of the most interesting valuable materials in clinical proteomics. Various technologies have been applied to analyze the urine proteome. Among them, mass spectrometry (MS)-based platforms have emerged as one of the most popular platforms for urinary protein and peptide identification. A large number of previous studies on the urine proteome mainly focused on the renal injury, but recent data show that analysis of the urinary proteome can also be highly meaningful on non-urogenital diseases. For example, urine proteomics has been applied in biomarker studies for hepatocellular carcinoma caused by hepatitis B virus, acute appendicitis and medulloblastoma [7–9].

In this study, we investigated the proteome expression profile in mouse urine infected with *T.* *gondii* using a label-free LC–MS/MS method in order to understand the functional changes during toxoplasmosis progression. We found that the majority of the differentially expressed proteins (DEPs) were upregulated after *T. gondii* infection and involved in disease and immunity related pathways. Mice in acute infection stage exhibited different proteome expression profiles than did mice in control and chronic infection groups. Besides, selected proteins were validated using parallel reaction monitoring (PRM) assay, which guaranteed the reliability of data obtained in this study. Our findings will help understand the mechanisms underlying renal histopathological changes during *T. gondii* infection and should aid exploration of novel biomarkers for toxoplasmosis.

## Methods

### Ethical approval

This study was approved by the Research Ethics Committee of Shandong University (ECSBMSSDU2020-2-027). All mice were cared for and handled in strict accordance with the regulations of Good Animal Practice requirements of the Animal Ethics Procedures and Guidelines of the People’s Republic of China for animal experimentation. All necessary measures were taken to minimize animal suffering and to reduce the number of animals used in this study.

### Mouse infection and urine collection

**Fig. 1:**
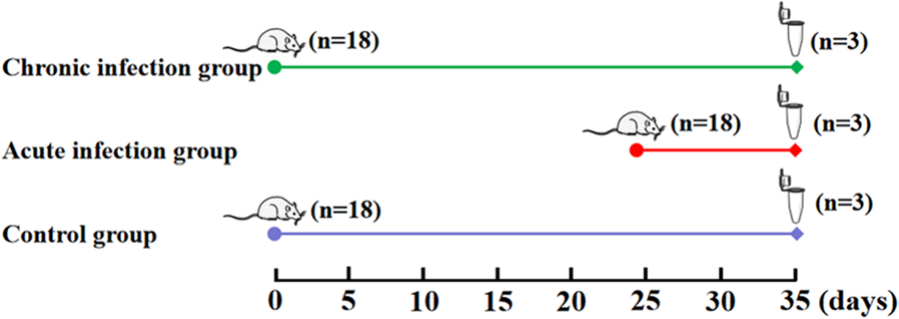
A schematic diagram showing the experimental design and urine sample collection. Circles indicate the time points of oral infection with *T.* *gondii* cysts, and diamonds indicate time points of urine sample collection

Six- to eight-week-old female BALB/c mice were purchased from Shandong University, China. The mice were placed in an environment with appropriated temperature and ventilation, 12 h of light and 12 h of darkness, with ad libitum feeding. In the label-free proteomics study, 54 mice were equally divided into three groups, namely acutely infected (AI) group, chronically infected (CI) group and control (Con) group. The urine sample collection strategy is shown in Fig. 1. Briefly, mice in AI and CI groups were orally administered 10 tissue cysts of *T.* *gondii* Prugniaud (Pru) strain that were dissolved in 100 µL phosphate buffered saline solution (PBS, pH 7.2). Control mice were orally administered the same dose of PBS. After 11 days of infection, infected mice showed typical symptoms of acute toxoplasmosis, and 24-hour urine samples were collected from the AI group. All infected mice returned to normal but showed losses in body weight after 35 days of infection, and 24-hour urine samples were collected from the CI group. Three biological repetitions were included in each group, and each biological repetition contained urine collected from six mice. In the liquid chromatography-parallel reaction monitoring-mass spectrometry (LC-PRM-MS) analysis, another 54 BALB/c mice were used, and samples were collected under the same protocols used in the above label-free proteomics study. Urine samples were centrifuged at 3000×*g* for 10 min at 4 °C to remove cellular debris. Supernatants were collected and frozen immediately in liquid nitrogen and stored at −80 °C until further use.

### Histopathological kidney studies

Kidneys were fixed in 10% neutral buffered formalin buffer for 2 weeks and then were dehydrated by gradually soaking in alcohol and xylene, and embedded in paraffin. Five-micrometer-thick sections were stained with hematoxylin and eosin (H&E) and examined under a digital optical microscope.

### Protein extraction and LC–MS/MS analysis

Urine samples melted on ice and their protein concentrations were determined using Bradford methods. After the addition of dithiothreitol (DTT) with the final concentration of 10 mM, urine samples were immersed into a water bath for 1 h at 56 °C. The samples were kept at room temperature in the dark room, and 55 mM iodoacetamide (IAM) (final concentration) was added and incubated for 1 h in order to block the cysteines. Samples were precipitated with acetone at a ratio of 1:4 v/v, incubated at −20 °C for more than 2 h and centrifuged at 12,000×*g* for 15 min at 4 °C. The supernatant was removed, and the pellet was washed twice with precooled acetone and centrifuged at 12,000×*g* for 30 min at 4 °C. The supernatant was discarded, and the pellet was air-dried and then resuspended in dissolution buffer which contained 0.1 M triethylammonium bicarbonate (TEAB, pH 8.5) and 6 M urea. To decrease the concentration of high-abundance proteins, the ProteoMiner™ protein enrichment kit (Bio-Rad) was used. The protein concentration was measured by the BCA Assay Kit (Thermo Fisher Scientific, Rockford, IL, USA) according to the manufacturer's instructions in triplicate for each sample. Equal amounts of proteins from each sample were digested with Trypsin Gold (Promega) at an enzyme-to-protein ratio of 1:50 at 37 °C overnight. Samples were desalted with C18 cartridge to remove the high urea, and desalted peptides were dried by vacuum centrifugation. Peptide samples were dissolved in buffer A (0.1% FA in water) and analyzed in an LC–MS/MS system that consisted of a an EASY-nLC™ 1200 UHPLC system (Thermo Fisher Scientific, Rockford, IL, USA) coupled with an Orbitrap Q Exactive HF-X mass spectrometer (Thermo Fisher Scientific, Rockford, IL, USA). Samples were loaded on a trapping column (75 µm × 2 cm, 3 µm, C18), and then separated through a homemade analytical column (15 cm × 150 µm, 1.9 µm). Samples were eluted with buffer B (buffer B: 0.1% FA in 80% ACN; buffer A: 0.1% FA in water) at 600 nL min^−1^ flow rate for 60 min. The separation gradient was set as follows: 5–10% B, 2 min; 10–30% B, 49 min; 30–50% B, 2 min; 50–90% B, 2 min; 90–100% B, 5 min.

A Q-Exactive HF-X mass spectrometer was operated in positive polarity mode with a spray voltage of 2.3 kV and capillary temperature of 320 °C. The data were acquired in the data-dependent mode. Survey full-scan MS spectra ranged from m/z 350–1,500 with a resolution of 60,000 at m/z 200. MS/MS spectra were acquired in higher-energy collisional dissociation (HCD) mode at a resolution of 15,000 (at 200 m/z) with an automatic gain control (AGC) target value of 1 × 10^5^, a maximum ion injection time of 45 ms, a normalized collision energy of 28%, an intensity threshold of 2.2e4 and the dynamic exclusion parameter of 20 s.

### Mass data analysis

The MS/MS data were acquired in raw files. Peak lists were searched against the Uniprot *Mus musculus* database (*Mus musculus*, 2016_06, 77,105 sequences, http://www.uniprot.org/) using Proteome Discoverer 2.2 (PD 2.2, thermo). The following parameters were applied: fixed modifications: carbamidomethyl (C); variable modifications: acetyl (protein N-term) and oxidation (M); MS/MS tolerance (FTMS): 10 ppm; fragment mass tolerance: 0.02 Da; type of quantification: precursor quantification; enzyme: trypsin; precursor abundance based on: intensity; max. missed cleavage sites: two; peptide-spectrum match (PSM) false discovery rate (FDR): 0.01; protein FDR: 0.01.

### Bioinformatics analysis

In order to characterize the function of the DEPs identified in this study, gene ontology (GO) analysis was performed (http://www.geneontology.org/) [10]. The *P* value of the GO term enrichment represented the significance of the genes annotated to a particular GO term, and significant GO enrichment was defined as *P* < 0.05. Pathway analyses of DEPs were performed through the KEGG database (KEGG, http://www.genome.jp/kegg/) [11]. The protein–protein interaction (PPI) networks associated with these proteins were retrieved from a web-based tool STRING database (https://string-db.org/cgi/input.pl) [12].

### Parallel reaction monitoring (PRM) analysis

Selected DEPs from the above label-free proteomics were further verified by LC-PRM/MS analysis. PRM analysis was performed on a Q Exactive mass spectrometer coupled with the EASY-nLC™ 1200 UHPLC system (Thermo Fisher). The same amounts of tryptic peptides of each sample were spiked with the equivalent heavy isotope-labeled peptide DSPSAPVNVT**V**R (bold V for heavy isotope labeling, serving as an internal standard), and injected into the trap column. Solvent A was 0.1% formic acid, and solvent B was 0.1% acetonitrile formic acid (80% acetonitrile). The peptides were balanced with 95% buffer A (0.1% formic acid) and then separated by a homemade analytical column with a linear gradient solution B (0.1% formic acid in 80% acetonitrile) for 1 h at a flow rate of 600 nL/min.

After that, MS was analyzed with the following parameters: full MS scan from 350 to 1500 m/z was acquired with an Orbitrap resolution of 60,000 (at *m/z* 200). The AGC value was set to 3 × 10^6^, and the maximum ion injection time was set to 20 ms. After a full MS scan, the precursors of highest abundance were selected for further MS/MS analysis. The AGC target value was set to 5 × 10^4^, and the maximum ion injection time was set to 80 ms. The Orbitrap resolution was set to 30,000 (at *m/z* 200). Ions were fragmented through higher-energy collisional dissociation (HCD) with normalized collision energies of 27 eV. Raw data were analyzed via Skyline software (MacCoss Lab, University of Washington, USA).

### Statistical analysis

Data from quantitative experiments were analyzed by Mann–Whitney test, and *P* values < 0.05 were deemed statistically significant. Protein features were considered to be significantly changed between different urine samples using a statistical *P* value < 0.05 and a fold change ≥ 1.5 or ≤ 0.67.

## Results

### Proteomic characterization

**Fig. 2:**
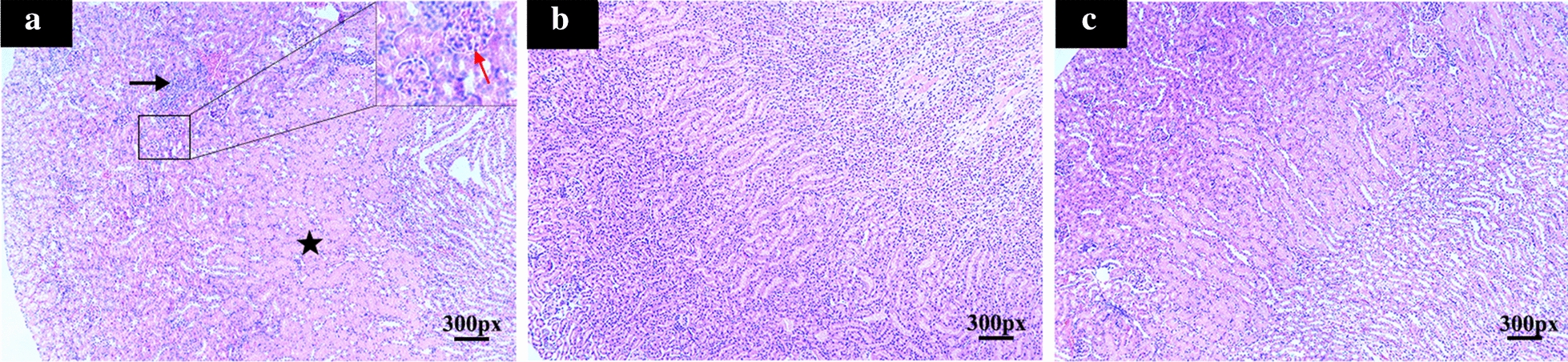
Histopathological changes in mouse kidneys infected with *Toxoplasma gondii *Pru strain. **a** A section of kidney from a mouse in acute infection group. The black arrow indicates lymphocytic mononuclear inflammatory infiltrate. The pentagram indicates tubular degeneration. The red arrow shows the proliferation of endothelial cells and neutrophil infiltration within the glomeruli. **b** Kidney histology of infected mice in the chronic infection group. **c** Kidney histology of control mice without any pathological changes

Mice in the AI group showed typical clinical symptoms of acute toxoplasmosis, including body weight loss, fever, anorexia, edema and messy hair. However, mice in the CI group recovered and showed normal daily food and water intake. Using H&E staining, kidney samples were examined for histopathological damages caused by *T. gondii *infection*.* As shown in Fig. 2a, infected mice at acute infection stage had acute nephritis, which was characterized as degenerated tubules, lymphocytic mononuclear inflammatory infiltrate and proliferation of glomerular endothelial cells. In contrast, mice in the chronic infection and control groups did not show any obvious pathological changes (Fig. 2b, c).

**Fig. 3:**
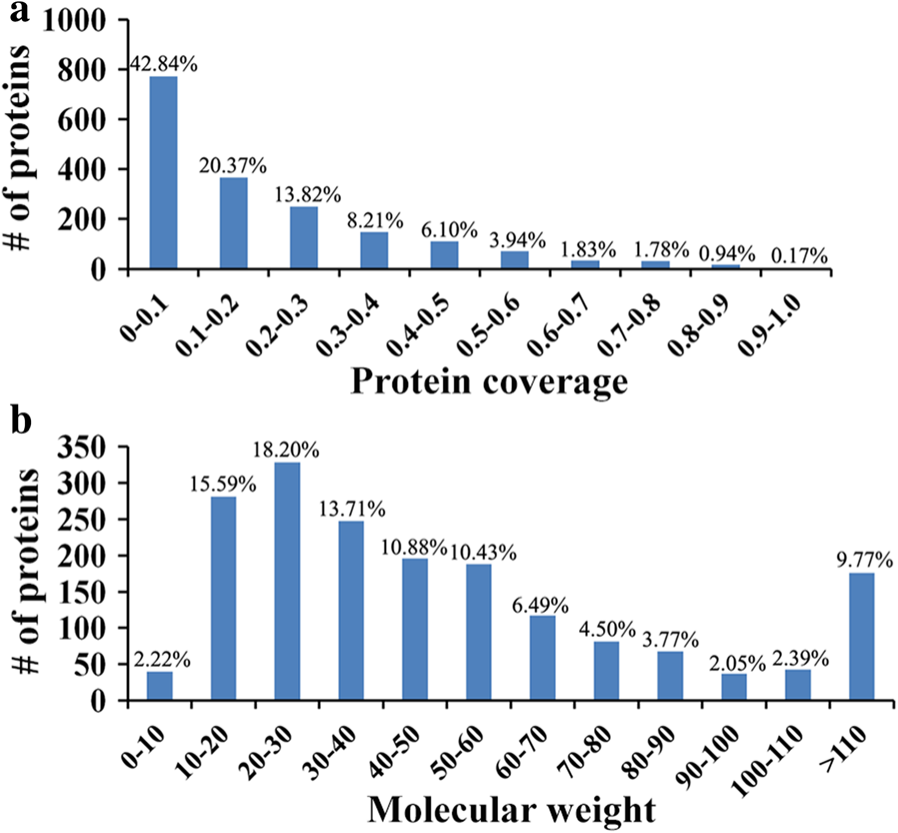
Features of urine proteins identified in this study. **a** Protein coverage. The X axis represents protein coverage, and the Y axis represents the number of identified proteins. **b** The molecular weight distribution. The X axis represents the molecular weight of the identified proteins, and the Y axis represents the number of proteins

**Fig. 4:**
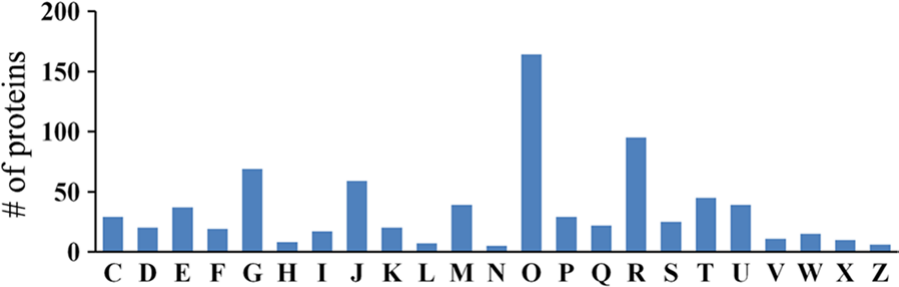
Cluster of Orthologous Group (COG) analysis of identified proteins from mouse urine. The vertical axis represents the number of proteins. The horizontal axis represents different COG classes. *C* Energy production and conversion, *D* cell cycle control, cell division, chromosome partitioning, *E* amino acid transport and metabolism, *F* nucleotide transport and metabolism, *G* carbohydrate transport and metabolism, *H* coenzyme transport and metabolism, *I* lipid transport and metabolism, *J* translation, ribosomal structure and biogenesis, *K* transcription, *L* replication, recombination and repair, *M* cell wall/membrane/envelope biogenesis, *N* cell motility, *O* posttranslational modification, protein turnover, chaperones, *P* inorganic ion transport and metabolism, *Q* secondary metabolites biosynthesis, transport and catabolism, *R* general function prediction only, *S* function unknown, *T* signal transduction mechanisms, *U* intracellular trafficking, secretion and vesicular transport, *V* defense mechanisms, *W* extracellular structures, *X* mobilome, prophages, transposons, *Z* cytoskeleton

In order to profile the host urine proteome during toxoplasmosis progression, a powerful label-free quantitative proteomics analysis was performed, and a total of 491,217 spectra were obtained. According to the Proteome Discoverer 2.2 searching, 13,414 high-scoring unique peptides were obtained, and 1802 proteins were identified. As shown in Fig. 3a, less a tenth of identified proteins have more than 50% protein coverage, and 42.84% of identified proteins have less than 10% coverage. The majority of these identified proteins were 10–60 kDa in weight (Fig. 3b). These proteins were then assigned to functional Cluster of Orthologous Group (COG) categories. As shown in Fig. 4, 732 identified proteins were divided into 23 categories, and “posttranslational modification, protein turnover, chaperones,” “general function prediction only,” “carbohydrate transport and metabolism” and “translation, ribosomal structure and biogenesis” were the four categories with the most identified proteins. Additionally, functional analyses of these identified proteins are shown in Additional file 1: Fig. S1.

### Urine proteome profiles during toxoplasmosis progression

**Fig. 5:**
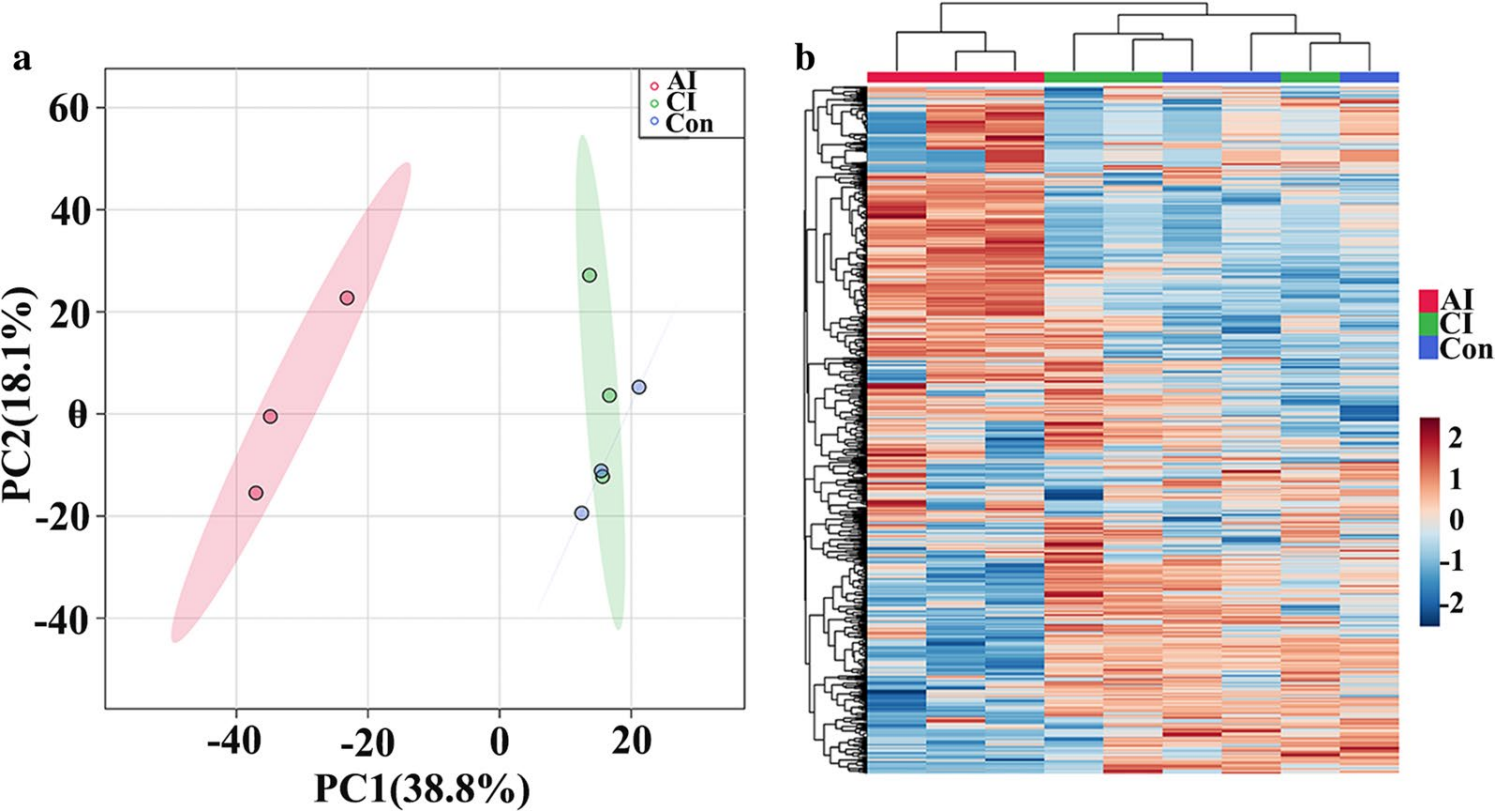
Differential proteomic profiles of mouse urine during *T.* *gondii* infection. **a** Principal component analysis (PCA) score scatter plots of proteins obtained from LC–MS/MS fingerprints. **b** Unsupervised hierarchical clustering of protein profiling data. Protein intensity is normalized so that blue represents low intensity and reddish color represents high intensity. Columns were hierarchically clustered based on a complete linkage using Pearson correlation coefficients as the distance measure. Sample groups including acutely infected, chronically infected and control are labeled as AI, CI and Con, respectively

To further characterize the urine proteome profile that might reveal a common response to *T.* *gondii* infection, we compared the proteomic data across the different mice groups by using two-dimensional principal component analysis (PCA). As shown in Fig. 5a, acutely infected mice were clearly differentiated from chronically infected and control mice. However, samples from chronically infected and control groups were intermixed, and the score plots did not show a good separation. Meanwhile, clustering of the proteomic profile is shown in Fig. 5b. Heat maps were constructed based on normalized protein abundance and showed that the proteomic profile in acutely infected group was distinguishable compared to the other two groups.

**Fig. 6:**
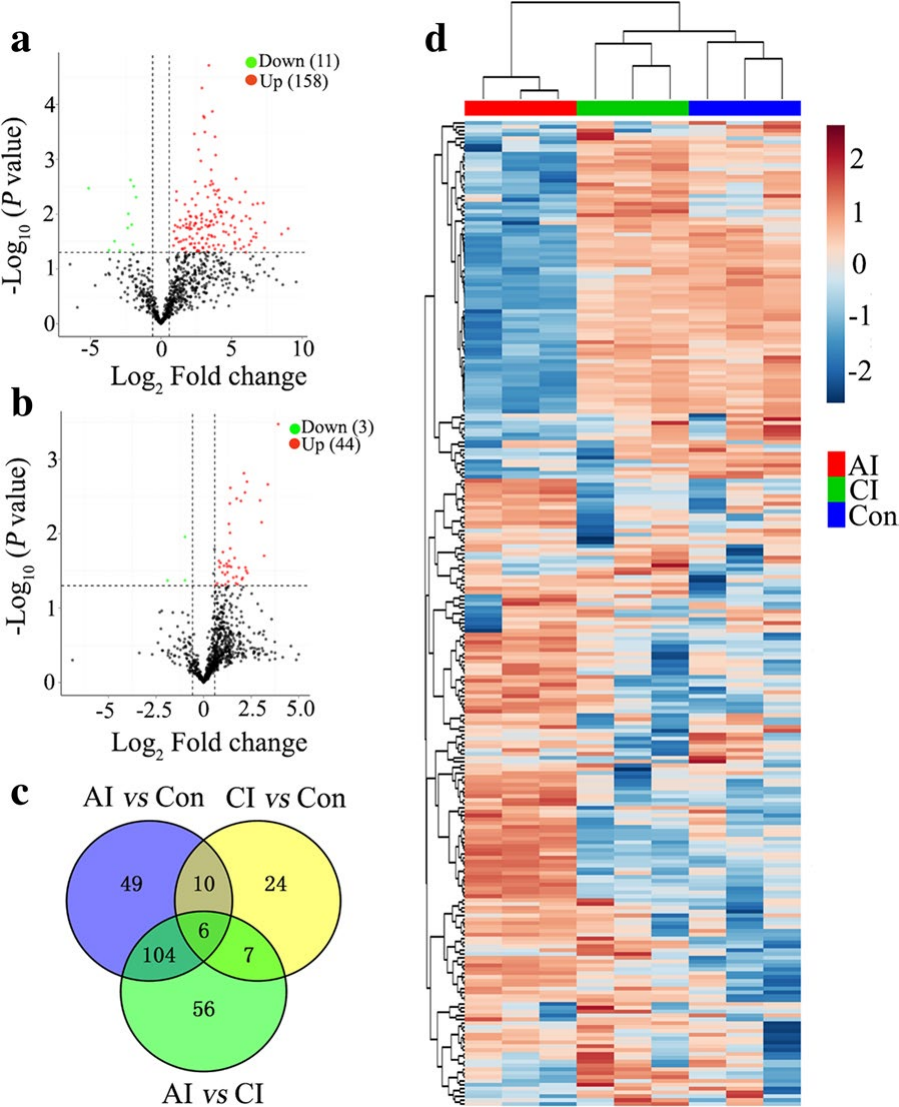
Summary of the differentially expressed proteins (DEPs) identified in mouse urine in response to *T.* *gondii* infection. **a** Volcano plots showing *P* values (–log_10_) versus protein ratios between acutely infected (AI) and control (Con) mice (log_2_). **b** Volcano plots showing *P* values (–log_10_) versus protein ratios between chronically infected (CI) and control mice (log_2_). **c** Venn diagram of the DEPs identified among AI, CI and Con groups. **d** Heat map of DEPs among all samples. Unsupervised hierarchical clustering was performed using the Pearson correlation as distance measure and average-linkage for linkage analysis. Brown and blue indicate higher and lower abundance, respectively

Next, we identified differences in protein expression among all groups. Compared to control group, 169 DEPs in the acute infection group were detected, among which 158 proteins were upregulated, while 11 proteins were downregulated (Fig. 6a). In the chronic infection group, 47 DEPs were identified with 44 upregulated and 3 downregulated (Fig. 6b). As shown in Fig. 6c, among these dysregulated proteins, 16 were common between AI vs. Con and CI vs. Con. Except for 12 major urinary protein precursors, all the DEPs found in both acute and chronic infections were upregulated (Table 1). The clustering of protein expression profiles derived from 256 DEPs in all samples is shown in Fig. 6d, and the tree shows a very good separation among all groups.

**Table 1:** List of dysregulated proteins identified throughout the entire infection process

Protein ID	Protein name	AI vs. Con		CI vs. Con	
		FC	*P* value	FC	*P* value
NP_001002898.1	Signal-regulatory protein beta 1	3.67	1.78E–02	4.51	2.79E–03
NP_001103968.1	Interferon alpha/beta receptor 2 isoform b precursor	2.21	2.77E–02	2.64	1.58E–02
NP_001137161.1	Histocompatibility 2, Q region locus 4 precursor	28.70	5.09E–03	10.38	2.17E–03
NP_001186924.1	Major urinary protein 12 precursor	0.13	4.62E–02	0.27	4.27E–02
NP_001230031.1	Hemolytic complement-like isoform 2 precursor	2.99	1.63E–02	2.59	3.93E–03
NP_031478.1	Thioredoxin-dependent peroxide reductase, mitochondrial precursor	11.79	1.55E–03	2.52	2.32E–02
NP_032989.2	Prostaglandin-H2 D-isomerase precursor	2.14	5.62E–03	1.85	2.47E–02
NP_034521.1	H-2 class I histocompatibility antigen, Q10 alpha chain precursor	7.49	5.01E–05	2.35	2.76E–02
NP_034875.1	Lymphocyte antigen 86 precursor	5.25	3.43E–02	2.32	2.53E–02
NP_035212.2	Polymeric immunoglobulin receptor precursor	7.66	2.71E–02	4.13	3.92E–02
NP_035280.1	Galectin-3-binding protein precursor	4.38	1.11E–02	4.33	1.54E–03
NP_059066.1	Haptoglobin isoform 1 preproprotein	16.78	8.22E–03	3.32	3.37E–03
NP_080887.1	Ribonuclease T2-B precursor	7.10	1.07E–03	2.02	1.77E–02
XP_011246439.1	PREDICTED: carbonic anhydrase 1 isoform X1	4.69	2.80E–02	2.10	2.70E–02
XP_017168039.1	PREDICTED: cadherin-13 isoform X2	2.93	4.30E–02	1.98	3.32E–02
XP_017174460.1	PREDICTED: H-2 class I histocompatibility antigen, K-W28 alpha chain isoform X10	37.75	1.77E–02	15.15	3.37E–04

### Biological functional analysis of DEPs

**Fig. 7:**
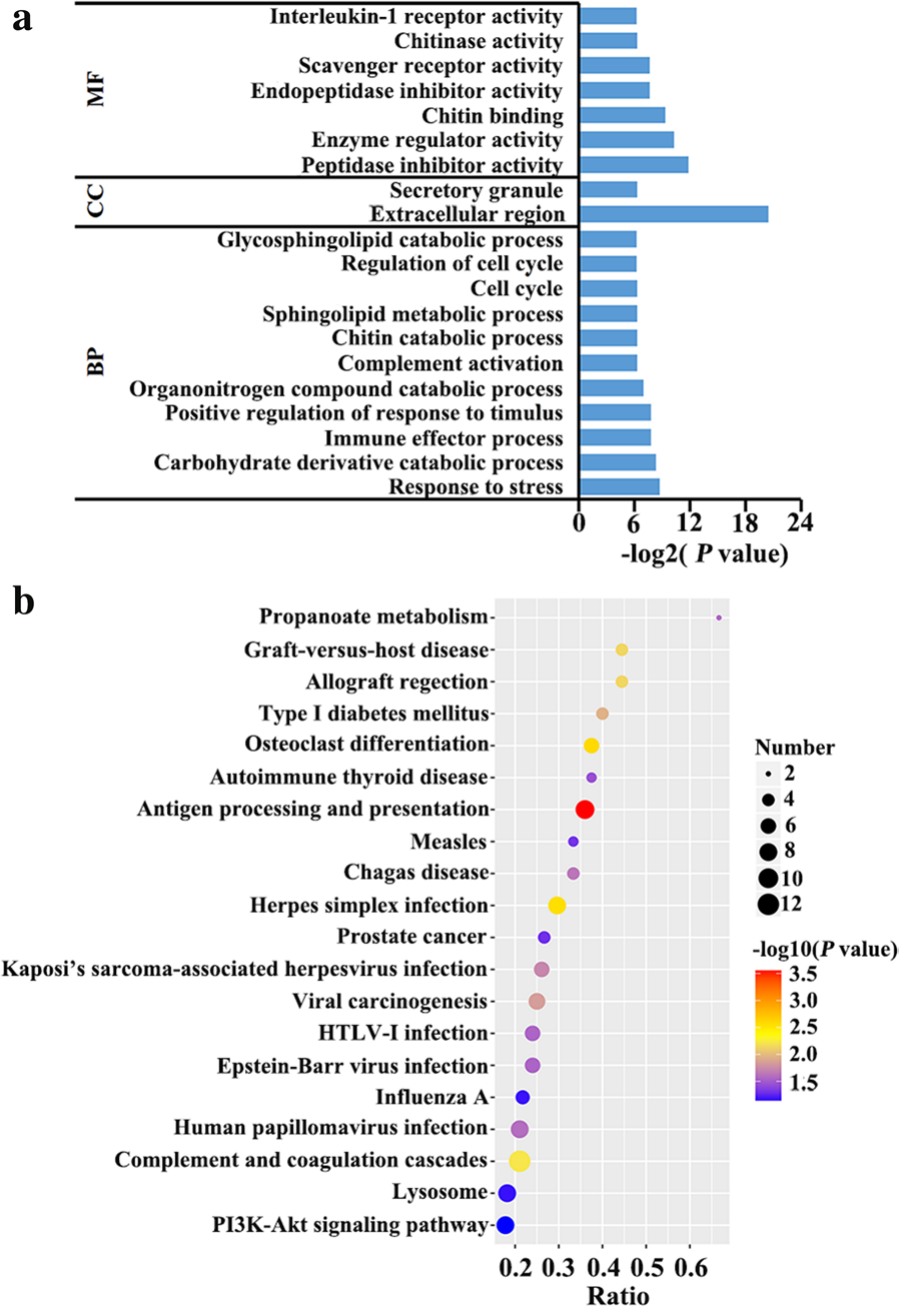
Functional enrichment analysis of the DEPs. **a** Gene ontology (GO) analysis of DEPs. The X-axis label denotes the number of DEPs, whereas the Y-axis label represents the corresponding GO terms. **b** The top 20 significantly enriched KEGG pathways of the DEPs. The X-axis label shows the rich factor. The Y-axis label shows the KEGG pathway terms. The color of the dots represents log_10_ (*P* value), and the size of the dot represents the number of DEPs enriched in the pathway

**Fig. 8:**
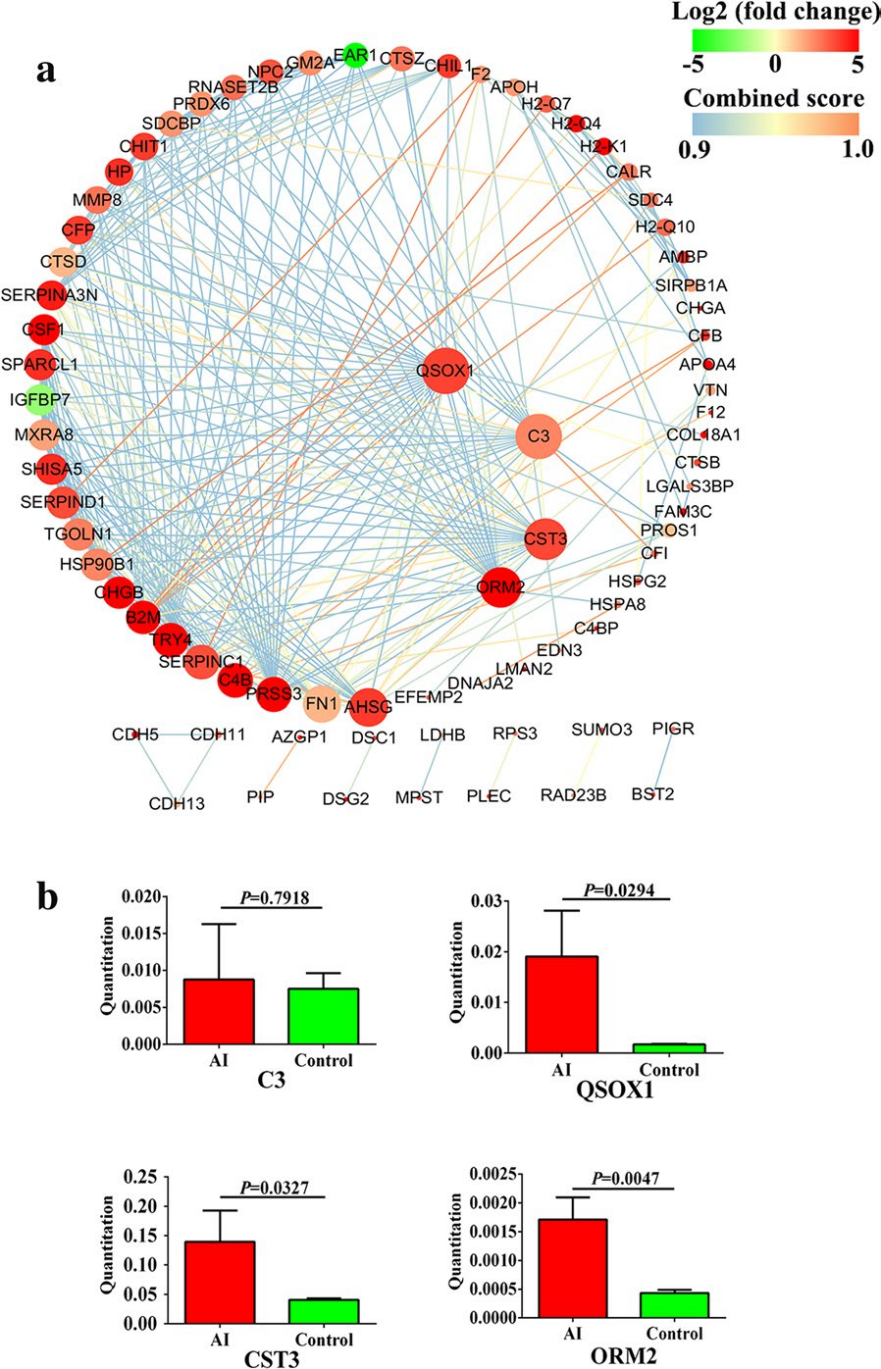
Protein–protein interaction network analysis. **a** The network consists of 77 DEPs identified between the acutely infected and normal mouse groups. The red node indicates upregulated and the green node indicates downregulated proteins (log_2_ fold change). The node size indicates high interaction degree (large) or low degree (small). Proteins that are associated to each other are linked by an edge. The color of the edge indicates the combined interaction score for a particular protein pair. **b** Validation of DEPs by PRM analysis

To better understand the roles of these significantly altered proteins, DEPs were analyzed by using web-based GO software (http://www.geneontology.org). The GO project includes three main modules: biological process, cellular component and molecular function. As shown in Fig. 7a, the top 20 most enriched GO terms are listed, and “response to stress,” “extracellular region” and “peptidase inhibitor activity” are the most prominent GO terms in each module between acutely infected mice and controls. In the comparison between chronically infected and control groups, “nuclease activity” under the molecular function module and “immune response” in the biological process module were the most prominent GO terms (Additional file 2: Fig. S2A). To understand the biological pathways that were affected during infection, DEPs were mapped to terms in the KEGG database. The top 20 highly enriched pathways in acute infection are shown in Fig. 7b, and include “antigen processing and presentation,” “osteoclast differentiation” and “herpes simplex infection.” In the comparison between chronically infected and controls, DEPs were enriched in disease-related and metabolic pathways (Additional file 2: Fig. S2B). The protein–protein interaction (PPI) network of these DEPs was built based on STRING 11.0. In the comparison between acutely infected mice and controls, the PPI network contained 77 protein nodes and 318 edges (Fig. 8a). The top four highly connected DEPs which include sulfhydryl oxidase 1 (QSOX1), complement C3 (C3), cystatin-3 (CST3) and alpha-1-acid glycoprotein 2 (ORM2) were further validated using PRM assay. Except for C3, the expression levels of the above top connected DEPs are in agreement with the findings in the label-free proteomics analysis (Fig. 8b).

## Discussion

All warm-blooded animals including humans are susceptible to *T.* *gondii*. However, up to now, there have been no applicable biomarkers that can reliably detect toxoplasmosis. The polypeptide pattern produced by the body fluid and tissue of the host is not only used to monitor the ongoing organ function, but also potentially reflects some diseases. Among them, urine is considered to be a valuable biological fluid containing rich diagnostic information for specific diseases. Thus, with the fast advances in MS-based proteomics, urine has been used in the explorations of biomarkers of various diseases. Many previous omics studies on toxoplasmosis were mainly focused on blood or tissues, but seldom on urine [13–16]. Therefore, in this study, we performed MS-based urine proteomics with a label-free method, which offered a comprehensive dynamic profile of the urine proteome during the infection of *T.* *gondii.*

A number of studies have been carried out to analyze the urinary proteome in both humans and animals. For example, Marimuthu et al., Adachi et al. and Li et al. reported 1823, 1543 and 1310 proteins in human urine, respectively [17–19]. Gargan et al. identified 1010 proteins in mouse urine using a label-free proteomics method [20]. Combining these published data, there are more than 2500 proteins that have now been found in urine. In this study, we identified 1802 proteins that contained at least one unique peptide, which further expands the repository of urinary proteins. Regarding the subcellular locations of the proteins identified in this study, we found that the majority of the identified proteins were derived from extracellular regions and membranes. Among diverse biological functions of these identified proteins, metabolic activity and immune response are most prominent. It is worth noting that 95 identified proteins are involved in proteolysis, which indicates that the process of toxoplasmosis might be closely associated with some proteolytic events.

In the acute infection stage, the infected kidney showed pathological changes of nephritis, including degenerated tubules and mononuclear inflammatory infiltrate. Meanwhile, other organs also exhibited pathological changes that have been well documented in our previously study [21]. There were 169 DEPs found at this stage. To investigate the biological functions of DEPs, GO analysis was performed and revealed that the “extracellular region” and “secretory granule” were significantly enriched. A previous study showed approximately one third of urinary proteins originate from plasma circulating throughout body via blood filtration, whereas the rest are derived from tissues and cells in the kidney and the urinary tract [22]. Hence, changes in the urinary proteome can reflect the disturbance of the body’s homeostasis during toxoplasmosis, not just renal pathological changes. Through KEGG analysis, 82 DEPs were involved in KEGG pathways, and disease- and immunity-related pathways were most enriched, such as “antigen processing and presentation,” “osteoclast differentiation” and “herpes simplex infection,” which indicated that proteins significantly increased or decreased at this stage might underlie the pathogenesis of acute toxoplasmosis. Mice at chronic infection stage have restored their physical health and showed normal food intake. Compared with the acute infection stage, the number of DEPs in the urine declined sharply by 72% at the chronic phase, which might account for restoration of body homeostasis. However, 30 DEPs identified at this stage were involved in 74 KEGG pathways. It is noteworthy that the majority of these pathways were related to infection and immunity, which indicated that *T.* *gondii* infection could cause adverse long-term effect. Although 16 DEPs were identified at both infection stages, the majority of them were precursor proteins without defined functions. It is worth noting that the expression level of signal-regulatory protein beta1 (SIRPB1) was elevated at both phases. SIRPB1 is expressed in monocytes and dendritic cells and interacts with DNAX-activating protein of 12 kDa (DAP12) that contains a single basic lysine residue within the hydrophobic transmembrane domain. A series of biological activity such as tyrosine phosphorylation and mitogen-activated protein kinase activation will take place following the stimulation of SIRPB1 [23]. Interestingly, higher levels of IL-12 were detected in DAP12-deficient mice following microorganism infection [24]. IL-12 can stimulate interferon (IFN)-gamma synthesis by natural killer cells and T lymphocytes, and thus initiates the resistance to *T.* *gondii*, which indicates that the SIRPB1/DAP12 complex might play important roles in *T.* *gondii* infection. Furthermore, PPI analysis pointed out functional associations among 77 proteins. Sulfhydryl oxidase 1, complement C3, cystatin-3 and alpha-1-acid glycoprotein 2 were the four most highly connected DEPs in the network. Sulfhydryl oxidase 1, a catalyst of disulfide bond formation, catalyzes the oxidation of sulfhydryl groups in peptide and introduces disulfides into unfolded reduced proteins, which play crucial roles in many diseases, such as cancer and prion formation [25, 26]. Cystatin-3 (also named cystatin-C), a cysteine protease inhibitor, functions as an inflammatory factor and has been widely recognized as a novel biomarker to predict the kidney function [27]. Alpha-1-acid glycoprotein (also known as orosomucoid) functions as transport protein in the plasma [28]. Additionally, alpha-1-acid glycoprotein is an acute-phase protein and is significantly increased in cancers and inflammatory diseases, which is considered a prognostic tool for determining inflammatory status [29]. Of note, all of the four most highly connected DEPs were validated and characterized by PRM assay, and the results showed that the direction of the expression was consistent between label-free proteomics and PRM. However, fold change was not significant for complement C3 in the PRM assay.

## Conclusions

We firstly investigated the proteome expression profile in mouse urine infected with *T.* *gondii* using a label-free LC–MS/MS method in order to understand the pathogenesis during toxoplasmosis progression. We found that the majority of the DEPs were upregulated after *T.* *gondii* infection and involved in disease- and immunity-related pathways. Mice in acute infection stage exhibited different proteome expression profiles than did mice in control and chronic infection groups. Our findings provide important data that will help understand the mechanisms underlying renal histopathological changes during *T.* *gondii* infection and should aid exploration of novel biomarkers for toxoplasmosis.

## Supplementary Information

**Additional file 1: Figure S1.** Functional analysis of the urine proteins identified in this study.

**Additional file 2: Figure S2.** Functional enrichment analysis of the DEPs identified in the comparison between CI and Con. (a) Gene ontology (GO) analysis of DEPs. The X-axis label denotes the number of DEPs, whereas the Y-axis label represents the corresponding GO terms. (b)The top 20 significantly enriched KEGG pathways of the DEPs. The X-axis label shows the rich factor. The Y-axis label shows the KEGG pathway terms. The color of the dots represents log10 (P-value) and the size of the dot represents the number of DEPs enriched in the pathway.

## Data Availability

The mass spectrometry proteomics data have been deposited to the ProteomeXchange Consortium (http://proteomecentral.proteomexchange.org) via the iProX partner repository with the dataset identifier PXD022458 [30].
