# Supplementary figures and images for: Urine proteomics for profiling of mouse toxoplasmosis using liquid chromatography tandem mass spectrometry analysis
Source: Parasit Vectors. 2021 Apr 20;14:211. doi: 10.1186/s13071-021-04713-6 (PMC8056516; doi:10.1186/s13071-021-04713-6)

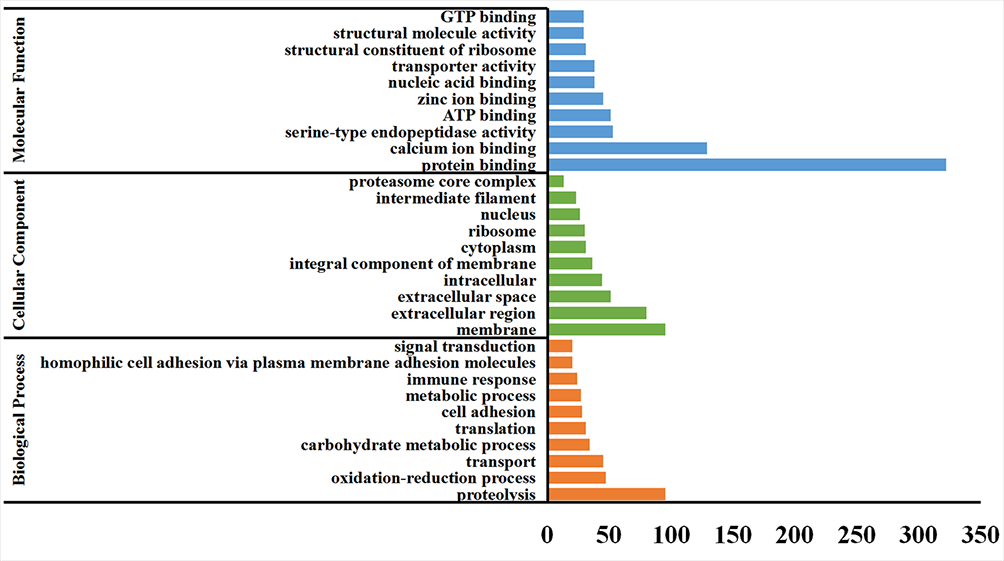

Supplement: Supplementary file 1 — Additional file 1: Figure S1. Functional analysis of the urine proteins identified in this study. [file 13071_2021_4713_MOESM1_ESM.tif]

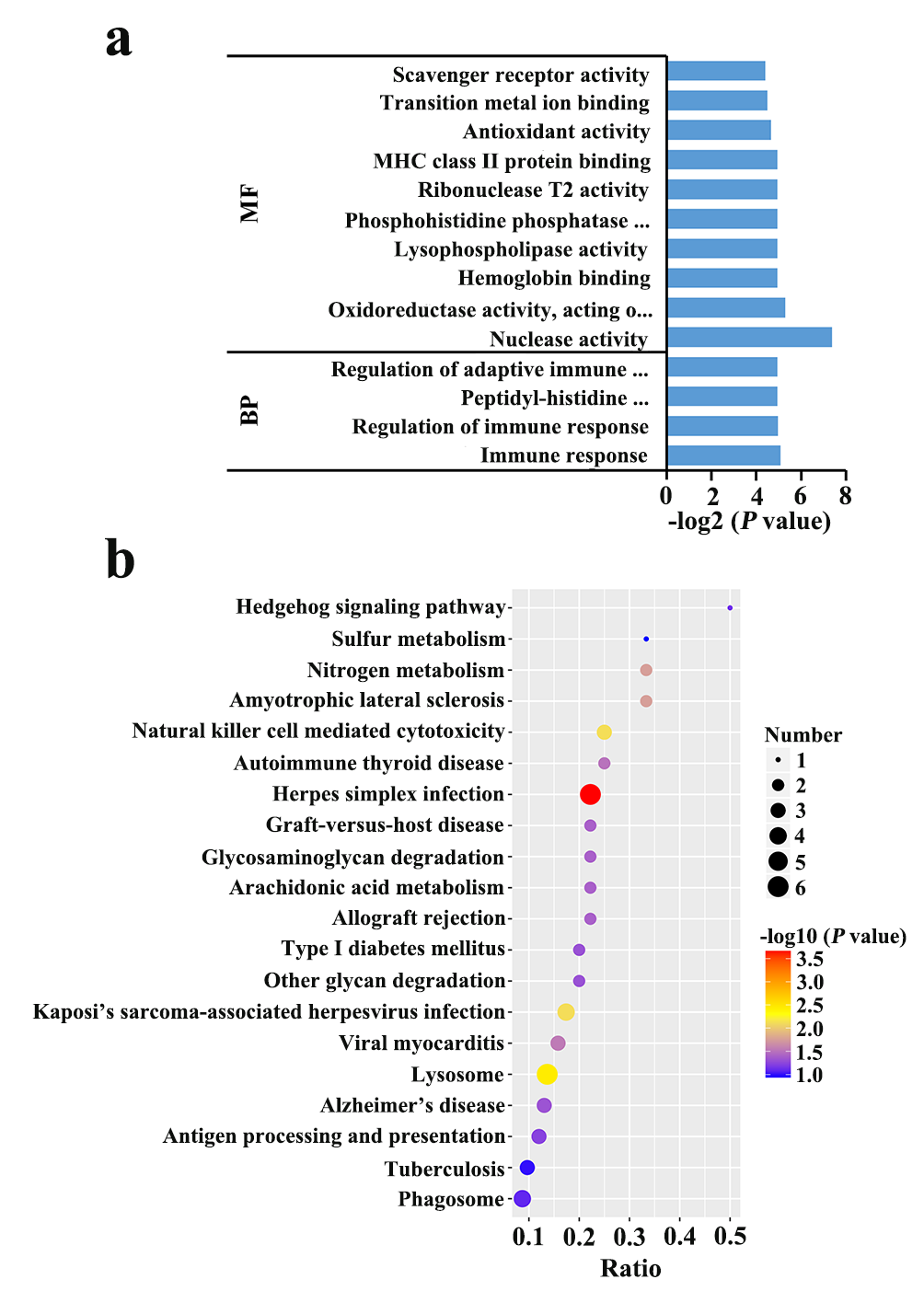

Supplement: Supplementary file 2 — Additional file 2: Figure S2. Functional enrichment analysis of the DEPs identified in the comparison between CI and Con. (a) Gene ontology (GO) analysis of DEPs. The X-axis label denotes the number of DEPs, whereas the Y-axis label represents the corresponding GO terms. (b)The top 20 significantly enriched KEGG pathways of the DEPs. The X-axis label shows the rich factor. The Y-axis label shows the KEGG pathway terms. The color of the dots represents log10 (P-value) and the size of the dot represents the number of DEPs enriched in the pathway. [file 13071_2021_4713_MOESM2_ESM.tif]
